# The Nature and Neural Correlates of Semantic Association versus Conceptual Similarity

**DOI:** 10.1093/cercor/bhv003

**Published:** 2015-01-30

**Authors:** Rebecca L. Jackson, Paul Hoffman, Gorana Pobric, Matthew A. Lambon Ralph

**Affiliations:** Neuroscience and Aphasia Research Unit (NARU), School of Psychological Sciences (Zochonis Building), University of Manchester, Manchester M13 9PL, UK

**Keywords:** fMRI, hub-and-spoke model, semantic memory, taxonomic, thematic

## Abstract

The ability to represent concepts and the relationships between them is critical to human cognition. How does the brain code relationships between items that share basic conceptual properties (e.g., dog and wolf) while simultaneously representing associative links between dissimilar items that co-occur in particular contexts (e.g., dog and bone)? To clarify the neural bases of these semantic components in neurologically intact participants, both types of semantic relationship were investigated in an fMRI study optimized for anterior temporal lobe (ATL) coverage. The clear principal finding was that the same core semantic network (ATL, superior temporal sulcus, ventral prefrontal cortex) was equivalently engaged when participants made semantic judgments on the basis of association or conceptual similarity. Direct comparisons revealed small, weaker differences for conceptual similarity > associative decisions (e.g., inferior prefrontal cortex) and associative > conceptual similarity (e.g., ventral parietal cortex) which appear to reflect graded differences in task difficulty. Indeed, once reaction time was entered as a covariate into the analysis, no associative versus category differences remained. The paper concludes with a discussion of how categorical/feature-based and associative relationships might be represented within a single, unified semantic system.

## Introduction

Investigating the nature of semantic representation has been a core pursuit in many different disciplines, including philosophy, linguistics, cognitive science, and neuroscience. The focus of the current study was on the comparison between, and neural basis of, 2 key forms of information that are extracted from semantic memory: associations and conceptual similarities (Lin and Murphy 2001; Crutch and Warrington 2005; Estes et al. 2011; Kalénine et al. 2012). The central question addressed in this study was: do semantic association and conceptual similarity arise from neuroanatomically separable components of semantic memory or are they the result of a single conceptualization process? In particular, we contrasted the alternative predictions made by the “dual-hub” theories of conceptualization (which propose a neural separation of semantic associations and conceptual similarity) versus the single “hub-and-spoke” framework (which suggests that these different aspects of semantic knowledge might be coded within a single framework).

Although there are lively and long-standing debates about the underpinning mechanisms, most researchers agree that concepts are formed from, and reflect a distillation of, our verbal and nonverbal experience (Wittgenstein 1953; Eggert 1977; Smith and Medin 1981; Barsalou 1999; Rogers et al. 2004; Lambon Ralph et al. 2010). Thus, for example, we know many things about the concept “croissant,” including features of its taste, smell, texture, visual form, knowledge of how it is made and served, etc. Semantic memory is, however, more than an exhaustive list of multimodal features. Crucially, we are able to extract higher order structures that code the relationships between concepts. First, “associative (or thematic) relationships” reflect the temporal and spatial co-occurrence of concepts, often contributing to the same acts or events. Thus, for example, croissants are associated with coffee and jam, despite these concepts having different appearances, tastes, smells, and functions. Second, we can generalize properties across concepts based on a sophisticated coding of “conceptual similarity.” For instance, we can correctly ascribe similar properties and actions to croissants, scones, crumpets, and naan bread, despite them having very different physical forms and occurring in different contexts. Both kinds of relationship types are central to the normal semantic cognition of adults (Lin and Murphy 2001). Many researchers have proposed different hypotheses on how these coherent, generalizable concepts are formed and, thus, this key dimension of semantic memory is given various theory-specific labels: family resemblances (Wittgenstein 1953); taxonomical/categorical similarity (Quillian 1968); prototypicality (Rosch 1975); feature-similarity (Smith and Medin. 1981; McRae and Cree. 2002). For the sake of brevity, the theory-neutral term conceptual similarity will be used hence forth.

The literature contains at least 3 types of inconclusive empirical comparisons of conceptual similarity versus associative relationships. Neuropsychological investigations potentially offer definitive information on the separability of these 2 forms of knowledge, if a double dissociation between associative and conceptual similarity could be established. Goldstein (1936, 1948) was perhaps the first to suggest a single dissociation in patients with semantic aphasia (Head 1926; Jefferies and Lambon Ralph 2006). Although not formally tested, Goldstein noted that these patients were able to detect and, perhaps were overly influenced by, strong associations. In addition, he noted that they found it difficult to consider the more abstract notion of categorically related items (a part of his broader notion of a loss of “abstract thinking” in semantic aphasia). A potentially related contrast was reported by Jefferies and Lambon Ralph (2006). One of a set of qualitative differences between semantic dementia (a neurodegenerative condition leading to atrophy focused on the anterior temporal lobe [ATL]) and semantic aphasia (a subtype of aphasia associated with prefrontal or temporoparietal lesions) is a difference in picture naming errors; semantic aphasia (SA) patients make a mixture of associative and category-related errors whereas standard deviation (SD) patients almost never produce associative semantic errors.

In an innovative study of aphasic picture naming, Schwartz et al. (2011) used voxel-based symptom–lesion mapping to relate the likelihood of each error type to lesion distribution. When focused on the relative rates of each error type, a higher rate of taxonomic errors was associated with voxel integrity in the ATL whereas more associative/thematic errors were predicted by lesions in temporoparietal cortex (TPC). Schwartz et al. (2011) concluded that there are separate stores for conceptual similarity (ATL) and associative relationships (TPC), a position that we refer to here as the “dual-hub model” (see also, Kalénine et al. 2012).

At least 3 neuropsychological phenomena do not seem to fit easily with this conclusion, however. First, all semantic aphasia patients make a mixture of category and associatively related errors and, thus, there is no absolute double dissociation within this group (Jefferies and Lambon Ralph 2006). Second, direct assessment has established that both semantic dementia and semantic aphasia patients are impaired at tasks requiring knowledge of associative relationships, such as the Camel and Cactus Test and the Pyramid and Palm Trees Test (Bozeat et al. 2000; Jefferies and Lambon Ralph 2006). Indeed, a direct comparison showed that semantic dementia patients were actually worse at identifying associative than conceptual similarity-based relationships, which appears incompatible with the notion that the ATL exclusively codes conceptual similarity-based relationships (Hoffman, Jones et al. 2013). Finally, previous investigations have suggested that the ATL and TPC regions may underpin different components of semantic cognition rather than different types of semantic representation (Jefferies 2013). Although it has not been established whether ATL regions are involved in both association and conceptual similarity, they appear to play a crucial role in the representation and extraction of semantic knowledge across modalities and categories (Patterson et al. 2007; Lambon Ralph et al. 2010).

In contrast, semantic aphasia patients with damage to the TPC or prefrontal cortex exhibit poorly controlled retrieval and manipulation of semantic memory rather than impaired representation per se (Jefferies and Lambon Ralph 2006). We refer to these executive functions as “semantic control.” This hypothesis is supported by evidence from fMRI and transcranial magnetic stimulation (TMS) studies suggesting that prefrontal cortex (PFC), posterior middle temporal gyrus (pMTG), and intraparietal sulcus may form a distributed control network (Thompson-Schill et al. 1997; Wagner et al. 2001; Badre and Wagner 2003; Duncan 2010; Whitney et al. 2011; Jefferies 2013; Noonan et al. 2013; Lambon Ralph 2014). There is currently minimal information to delineate the individual contribution of these regions in semantic control. One possibility is that the pMTG may be relevant for semantic control, whereas frontal and parietal regions may be involved in executive function regardless of domain (Jefferies 2013; Noonan et al. 2013). Alternatively, the pMTG may not be responsible for control per se, but act as an interface between temporal areas responsible for representation and frontoparietal control regions.

To clarify these issues, we used fMRI to contrast associative relationships and conceptual similarity directly, and also manipulated the level of semantic control. A second key novelty in this study was methodological, namely a strict separation and direct probing of associative versus conceptual knowledge. This is an important step in that many concepts are related in both ways (e.g., “cat” and “mouse”) and previous comparative investigations have been dogged by this issue. Within experimental psychology, semantic priming has been demonstrated for both associative and conceptual similarity-based relations and attempts to separate these different effects have led to conflicting results (Shelton and Martin 1992; McRae and Boisvert 1998; Cree et al. 1999; Lucas 2000; Hutchison 2003; Hare et al. 2009). Likewise, previous fMRI studies comparing the 2 types of semantic relationship have found different results, ranging from no difference to large-scale differences over both hemispheres (Kotz et al. 2002; Kalénine et al. 2009; Sass et al. 2009). This may be because the studies lacked appropriate stimuli for comparison, for instance adopting taxonomically related words that are also associated with each other (e.g., Kotz et al. 2002; Sachs et al. 2008) or using picture stimuli, which may encourage participants to focus on lower level perceptual similarities (e.g., Kalénine et al. 2009). Furthermore, most studies have not probed knowledge of conceptual similarity versus associative relationships but have relied upon implicit processing of these relationships via priming which requires the neuroimaging method to detect a small subtle behavioral effect (e.g., Sachs et al. 2008; Sass et al. 2009).

In order to explore these key theoretical questions and alternative hypotheses, this study was designed in the following manner. First, there was a direct manipulation of 2 factors: type of semantic relationship (associative vs. conceptual similarity) and semantic control (hard vs. easy decisions; see Table 1 for example stimuli). Second, knowledge of each type of relationship was directly probed in the same semantic decision task rather than relying on secondary, implicit activation. Finally, the fMRI data were acquired in a way that allows full coverage of the entire semantic network including the ventral aspects of the ATL. Various methodological issues in previous investigations have led to inconsistent coverage and sensitivity to activation in this region (Embleton et al. 2010). Accordingly, the present study utilized a dual-echo gradient echo planar imaging (EPI) paradigm to ensure ATL coverage (Halai et al. 2014).

**Table 1:** Example stimuli for each condition in the 3 tasks

	Probe	Target	Foil
Semantic judgment task			
Association	Vase	Tulip	Elephant
Conceptual similarity	Vase	Bucket	Platform
Baseline (letter matching) task			
Low control demands	##HΨz##	bqwcHΨz	ctkdLXQ
High control demands	##HΨz##	bqwcHΨz	cHΨdLXQ
Task to vary semantic control demands			
Low control demands	Mountain	Pyramid	Doe
High control demands	Mountain	Pyramid	Arch

Type of semantic relationship was varied in the main semantic judgment task and the necessary level of control was manipulated in the baseline letter matching task and separate similarity-based semantic task. In the 2 semantic tasks, participants chose the word most related to the probe word and in the baseline task participants chose the item with the most letters from the probe.

This study was designed to investigate 3 ideas. A strong version of the dual-hub model would predict selective TPC activation for the associative condition compared with conceptual similarity and selective ATL activation for the opposite contrast. A single hub theory such as the hub-and-spoke model would predict significant positive activation of the ATL in both conditions. Finally, an alternative explanation of Schwartz et al. (2011) results is a role of the TPC in some sort of semantic control process that may be more important for associative relationships but can be accounted for by a general measure of semantic difficulty.

## Materials and Methods

### Participants

Twenty-five healthy native-English speakers took part in the experiment (16 females, age range 20–42 years, mean age 25.48 years, SD 6.49 years). One was excluded due to low overall performance suggesting inattention/noncompliance (overall performance for this participant 65%, overall performance for other 24 participants 90%, *t*_(24)_ = 19.94, *P* < 0.001). All participants were strongly right handed, with a laterality quotient above 70 on the Edinburgh Handedness Inventory (Oldfield 1971) and had normal or corrected-to-normal vision. All participants gave informed consent and the study was approved by the local ethics board.

### Stimuli

A semantic judgment task employing trials based on either association or conceptual similarity was employed along with a letter matching task designed to provide a high-level baseline. A manipulation of semantic control was included in a separate set of conceptual similarity judgments. A manipulation of nonsemantic control was incorporated within the letter matching task. An example trial from each condition in each task is displayed in Table 1.

#### Semantic Judgment Task

Participants were presented with triads of concrete nouns and asked to judge which of the 2 options was more related to the probe word (for full list see Supplementary Table 3). The probe–target relationship was based on either conceptual similarity or association. Semantic associative strength was quantified using latent semantic analysis, a technique that represents relationships between words based on the degree to which they are used in similar linguistic contexts. Hoffman, Lambon Ralph et al. (2013) performed low associative strength (LSA) on the British National Corpus using the standard approach described by Landauer and Dumais (1997). This corpus includes more than 87 million words from 3125 different sources. A matrix was generated coding frequency of occurrence for each word in each context and single-value decomposition was applied to these data, yielding LSA representations for words based on their contextual similarity. Pairs of words with a relationship higher than 0.2 in the resultant LSA measure were considered associated and lower than 0.2 were not. In order to separate the 2 semantic measures, associative targets had to have very low levels of conceptual similarity, most commonly selected to be in a different domain (e.g., living vs. artifacts) or, if this was not possible, in a different superordinate category with a low number of shared features (e.g., tools vs. clothing). Conversely, conceptually similar targets were selected from the same semantic category but had very LSA (scores below 0.2). There was a large, significant difference between the probe–target LSA values for the associative (average = 0.474, SD = 0.182) versus conceptually similar trials (average = 0.045, SD = 0.076; *t* = 20.334, *P* < 0.001).The associated and conceptually similar targets were matched on CELEX frequency (associative mean = 28.91, SD = 44.82; conceptually similar mean = 29.53, SD = 54.50; *t*_(95)_ = −0.084, *P* < 0.5), Bristol/MRC imageability norms (associative mean = 567.69, SD = 62.82; conceptually similar mean = 569.08, SD = 63.95; *t*_(95)_ = −0.155, *P* < 0.5), letter length (associative mean = 5.44, SD = 1.72; conceptually similar mean = 5.54, SD = 1.72; *t*_(95)_ = −0.473, *P* < 0.5) and syllable length (associative mean = 1.7, SD = 0.7; conceptually similar mean = 1.68, SD = 0.76; *t*_(95)_ = 0.222, *P* < 0.5) taken from the NWatch program (Davis 2005).

Two trials were derived for each probe, an associative versus a conceptually similar trial, though individual participants only saw one version in the experiment (counterbalanced across participants). The targets for the associative trials were used as foils for the similar trials and vice versa, ensuring that the overall set of words was identical for the 2 conditions, reducing potential confounds. All foils had an LSA value lower than 0.2 with their respective probe and target items, and were from the same domain as the target. Foils in the conceptually similar trials were in a different superordinate category making them less conceptually similar than the targets. The LSA values for the probe—associative foils (mean = −0.007, SD = 0.059) were matched to those for the probe—conceptually similar foils (mean = 0.002, SD = 0.061; *t*_(95)_ = −1.223, *P* > 0.05). This meant that the foils in the associative condition were less associated to the probe than the target (*t*_(95)_ = −23.348, *P* < 0.001) but both were conceptually dissimilar.

Ninety-six associative and 96 conceptual similarity trials were created. The greater relatedness of the targets than foils to the probe item was confirmed via 1) similarity ratings on a 7-point scale from “not at all similar” to “highly similar” by 11 participants who did not take part in the fMRI study (*t*_(95)_ = 29.983, *P* < 0.001) and 2) a behavioral pilot of 9 participants (9 females, mean age 19.33 years, SD 1.0) which confirmed high accuracy on the task in both conditions (association – accuracy = 0.903, RT = 1248.25; conceptual similarity – accuracy = 0.887, RT = 1396.52).

#### Task to Vary Semantic Control Demand

A further 96 probe–target conceptually similar pairs were created in the same manner as the main task. Two different foils were combined with each target–probe pair. One foil was selected from an unrelated domain to the probe item in order to minimize the level of control necessary to reject the foil and select the target (e.g., barrel–box, combined with the foil, plum). The other foil was selected from the same domain and a related category to the target and probe (e.g., barrel–box, paired with the foil, seat) and, thus, greater control was needed in these trials (for full list, see Supplementary Table 5). Targets were matched to related and unrelated foils on frequency (high; *t*_(95)_ = , *P* > 0.5, low; *t*_(95)_ = , *P* > 0.5), imageability (high; *t*_(95)_=, *P* > 0.5, low; *t*_(95)_=, *P* > 0.5), LSA value with probe (high; *t*_(95)_ = 0.086, *P* > 0.5, low; *t*_(95)_ = 0.009, *P* > 0.5), letter length (high; *t*_(95)_ = −0.216, *P* > 0.5, low; *t*_(95)_ = 0.309, *P* > 0.5), and syllable length (high; *t*_(95)_ = 0.291, *P* > 0.5, low; *t*_(95)_ = −0.212, *P* > 0.5). Each participant completed the high control version for half of the trials and the low control version for the other half (counterbalanced across participants). Using the same rating system described above, participants confirmed greater semantic relatedness of probe–target than probe–foil in both high control (*t*_(95)_ = 17.294, *P* < 0.001) and low control conditions (*t*_(95)_ = 13.284, *P* < 0.001).

#### Baseline (Letter Matching) Task

The goal of this task was to provide a nonsemantic but challenging visual-matching baseline activity against which the semantic neuroimaging data could be compared. Participants were asked to indicate which of 2 mixed letter-symbol strings contained more letters in common with the probe string (for full list see Supplementary Table 4). Probes included a Greek letter, dissimilar to those found in the English alphabet, flanked by 2 English letters. Hash symbols were then placed either side to make the string 7 characters long. The target included the same Greek letter and one or both of the English letters found in the probe in the same order but at any position in a 7-letter string. As in the semantic control-varying task, each probe–target pair had 2 different foils to alter task difficulty and thus allow assessment of nonsemantic executive control. Low control foils did not include any of the same letters as the probe. High control foils included the Greek letter and one or 2 of the English letters from the probe. Each participant received half of the high and half of the low control foils (counterbalanced across participants). The behavioral pilot confirmed that participants were able to perform the task and that it was as challenging, in terms of RT and accuracy, as the main semantic tasks (accuracy = 0.856, RT = 1693.29). RT for the high control condition (average 2158.85, SD 500.35) was significantly longer than the low control condition (average 1993.48, SD 493.77, *t*_(23)_ = 4.793, *P* < 0.001).

### Procedure

Participants practiced 20 trials of each task outside the scanner. Nine trials from each task were then repeated in intermixed miniblocks to simulate the presentation during scanning. A further 9 were repeated inside the scanner. Miniblocks lasted 15 s and contained 3 trials from one condition. All tasks started with a central fixation cross presented for 1000 ms. In the first trial of each miniblock, a cue was presented above fixation to allow participants to prepare for the correct task reducing task-switching effects. For both semantic tasks, the cue was “WORDS,” for the letter matching task it was “LETTERS.” The stimuli were then presented for 4000 ms in Times New Roman at size 24. The probe was displayed in the top center with the 2 options on the left and right at the bottom of the screen. During this time, participants responded by pressing one of 2 buttons representing the left and right options.

There were 4 runs each lasting 10 min. Three contained the main task, letter matching (baseline) task and rest. One contained the semantic control-varying task and rest. The order of these was counterbalanced. A pseudorandomized order of miniblocks was employed. Presentation of individual trials was randomized within miniblocks. There were 32 miniblocks, each, for the semantic control task, letter matching task, rest, and the associative versus conceptually similar versions of the main task. The letter matching and semantic control-varying tasks included 16 high and 16 low control miniblocks.

### Imaging and Data Analysis

Scanning was performed with a Phillips Achieva 3.0T TX series system with 32-channel SENSE coil with a SENSE factor of 2.5. Within the scanner, participants wore noise-cancelling Mk II+ headphones (MR Confon, Magdeburg, Germany). A structural reference was obtained with an in-plane resolution of 0.938 and slice thickness of 1.173.

Two echoes were used in parallel. A short echo at 12 ms allows for reduced spin dephasing leading to less signal loss in areas of high magnetic susceptibility while a standard long echo at 35 ms maintains high contrast sensitivity throughout the brain. The use of multiple echoes has been shown to reduce signal dropout, particularly in inferior temporal and frontal regions (Poser and Norris 2007, 2009; Halai et al. 2014). Combining the echoes through linear summation has been shown to be optimal (Poser et al. 2006; Halai et al. 2014). Each run included 211 functional scans covering the whole brain with a field of view of 240 × 240 mm, resolution matrix of 80 × 80, TR of 2.8, flip angle of 85°, reconstructed voxel size of 3 mm and slice thickness of 4 mm. The field of view was tilted up to 45° off the AC–PC line to reduce ghosting of the temporal pole.

Analysis was carried out using statistical parametric mapping (SPM8) software (Wellcome Trust Centre for Neuroimaging). Functional images were realigned to the individual's first image using a rigid body transform in order to correct for motion artifacts. The functional images were then coregistered to the individual's anatomical scan. Spatial normalization to the Montreal Neurological Institute template was achieved using the DARTEL toolbox (Ashburner 2007) by group-wise registration of individual's gray and white matter to a template brain created from the group mean. This increases the registration between individuals from the standard SPM normalization allowing more accurate localization and greater sensitivity. Smoothing was performed using an 8 mm full-width half maximum (FWHM) Gaussian kernel. A general linear model was created with all conditions modeled as box car functions convolved with a canonical HRF (rest was modeled implicitly). A high-pass filter with a cutoff of 128 s was used.

In the first analysis step, the main semantic judgment task was contrasted with the letter matching task to reveal the areas involved in general semantic processing. Association and conceptual similarity trials were contrasted with the letter matching task, and were compared directly as well. Effects of semantic control demands were assessed by contrasting the high versus low conditions in the semantic control task. Nonsemantic control demands were assessed by contrasting high and low control trials in the baseline letter task. Whole-brain analyses were subjected to FWE correction at the cluster level with a critical cluster level of 0.05. In the second analysis step, a new model was created to assess the effect of reaction time on neural activity. This model included letter matching and semantic conditions, with semantic trials modulated by 1) RT and 2) association versus conceptual similarity. This allowed 1) assessment of which areas increase their activation for longer semantic decision times, using the RT regressor, and then 2) a direct comparison of the (association vs. conceptual similarity) contrast and influence of semantic decision times—allowing us to assess whether any brain areas are sensitive to the subtype of semantic knowledge once semantic RTs are accounted for. In this analysis, RT and relationship type regressors were entered simultaneously into the model in order to assess the unique variance of each factor (i.e., we turned off the default serial orthogonalization option in SPM8). This is preferable to assessing the effect of relationship type after taking out all the variance that could relate to RT, as this may bias the findings to relate to RT alone. Third, conjunction analyses in SPM were used to compare regions found to be significant for the conceptual similarity > association contrast 1) with the areas found to be more active with high RT throughout the semantic task and 2) with regions more active for high semantic control > low semantic control. This form of conjunction analysis is a stringent measure of overlap because a region is only highlighted if activation is significant after cluster correction in “both” contrasts. Finally, ROIs were created based on peak co-ordinates from previous studies (see Results) and analyzed in the MarsBar toolbox (Brett et al. 2002). ROIs were spheres with a diameter of 10 mm. Statistics were conducted on the mean activation of the voxels within the ROI.

## Results

### Behavioral Data

The conceptual similarity judgments (mean RT = 1783.69, SD = 277.44) had significantly longer reaction times than the associative trials (mean RT = 1653.68, SD = 286.48; *t*_(23)_ = −4.58, *P* < 0.05). As designed, the letter matching task was harder than both the conceptual similarity (mean RT = 2076.16, SD = 265.49; *t*_(23)_ = 7.33, *P* < 0.05) and association-related trials (*t*_(23)_ = 10.9, *P* < 0.05), thereby providing an appropriate a high-level control condition. The manipulations of control in both the semantic and letter matching tasks successfully led to significant differences in RT as expected (high semantic control [mean RT = 1939.16, SD = 273.71], low semantic control [mean RT = 1809.55, SD = 299.24]; *t*_(23)_ = 4.22, *P* < 0.05, high nonsemantic control [mean RT = 2158.85, SD = 500.35], low nonsemantic control [mean RT = 1993.48, SD = 493.77]; *t*_(23)_ = 4.79, *P* < 0.05)].

### Whole-Brain Analyses

**Figure 1.**
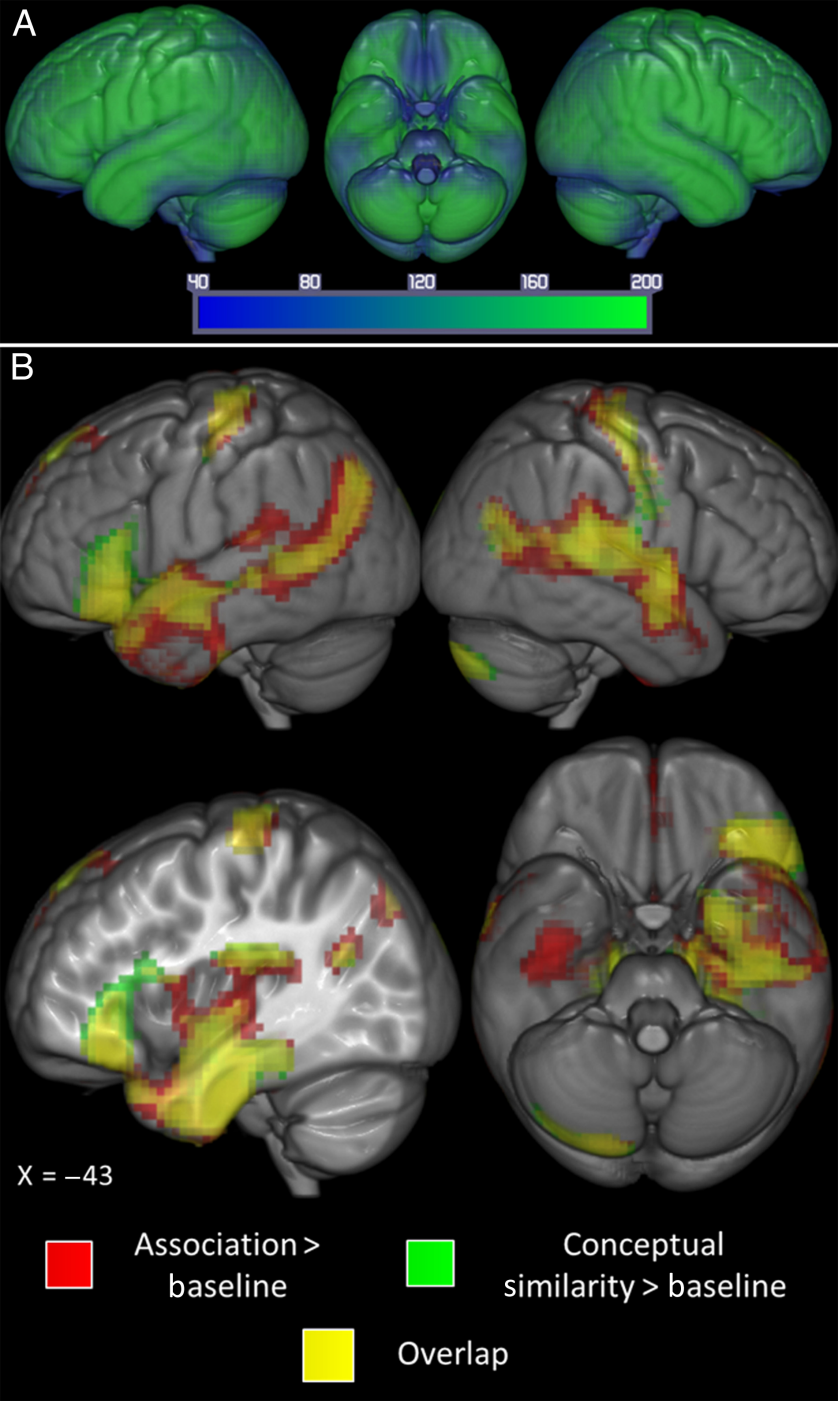
(*A*) Average temporal signal-to-noise ratio for the smoothed group echo planar imaging data in MNI space. The map is set at a threshold of 40, considered to be the minimum TSNR required to reliably detect differences in signal (Murphy et al. 2007; Simmons et al. 2010; Wang et al. 2013) and is displayed as a range from 40 (dark blue) to 200 (bright green). Use of the dual-echo technique meant signal reached the minimum threshold throughout the ATL and inferior frontal regions with some subregions far exceeding this with values above 200. (*B*) Significant activation for the contrasts association judgments > letter matching (red) and conceptual similarity judgments > letter matching (green); yellow = overlap. Voxels significant at 0.05 with an FWE correction at the cluster level with a critical cluster level of 0.05.

Average signal-to-noise ratio of the EPI data is displayed in Figure 1. This shows the high signal found with dual-echo EPI throughout the brain including in key inferior temporal and frontal regions.

### Semantic Task versus Letter Matching Task

All whole-brain analyses reported employ an FWE correction at the cluster level with a critical cluster level of 0.05 as well as significance at the voxel level of 0.001 and are reported in MNI space. Activation was first compared between the semantic and letter matching tasks. Areas of peak activation for the semantic task are summarized in Table 2. Both left and right temporal clusters extended across a large region to include the temporal pole, Heschl's gyrus, superior temporal gyrus (STG), middle temporal gyrus (MTG), inferior temporal gyrus (ITG), fusiform gyrus, hippocampus, parahippocampal gyrus, amygdala, insula, rolandic operculum, and cerebellum. Both clusters also extended posteriorly to mid-occipital cortex with activity in the angular gyrus on the left only. In addition, the cluster on the left included inferior frontal gyrus and mid-orbital frontal cortex. The activity centered round right STG also extended superiorly into the right pre- and postcentral gyri. Left pre- and postcentral gyri activation can be seen in a third cluster. Activation within the mid cingulum was bilateral and extended superiorly in to left and right supplementary motor area. The clusters within the cuneus and medial orbitofrontal cortex were also bilateral with activity extending from the frontal region inferiorly to the left rectus. Subtracting the semantic task from the letter matching task gave a large area of activation throughout bilateral occipital, parietal, and frontal lobes as well as the thalamic nuclei, right putamen, right insula, and bilateral posterior fusiform gyrus (see Table 2).

**Table 2:** Significant activation clusters for the contrast semantic task versus letter matching task

Contrast	Region of activation	Cluster extent (voxels)	Max *z* value	*P* value (FWE corrected)	Peak region	Peak MNI coordinate		
						*X*	*Y*	*Z*
Semantic > letter matching	R temporal	3436	7.02	>0.001	R STG	60	3	−3
					R STG	45	−3	−15
					R calcarine	27	−48	9
	L temporal	5630	7	>0.001	L PHG	−21	−21	−21
					L MTG	−45	−15	−12
					L ITG	−45	−15	−27
	L precentral gyrus	233	5.87	>0.001	L precentral	−45	−18	63
					L precentral	−33	−21	72
	Cerebellum	369	5.65	>0.001	R cerebellum	21	−84	−36
	L medial frontal	388	5.14	>0.001	L superior MFL	−9	48	39
					L superior MFL	−9	54	30
					L superior MFL	−9	57	18
	Cingulate	166	4.78	0.003	R mid cingulum	12	−3	45
					R mid cingulum	3	3	39
					R mid cingulum	0	−12	48
	L OFC	192	4.71	0.002	L medial OFC	−3	54	−12
					L anterior cingulum	−15	42	−3
	Cuneus	160	4.4	0.004	R cuneus	9	−84	27
					L cuneus	−3	−84	27
Letter matching > semantic	L occipitoparietal cortex	12 659	7.76	>0.001	L inferior occipital	−30	−75	−9
					L IPL	−42	−39	42
					L posterior FG	−39	−66	−12
	L inferior frontal	3975	7.06	>0.001	L precentral	−30	−3	45
					L IFG	51	9	27
					L IFG	42	9	30
	L mid frontal	636	5.91	>0.001	L MFG	−39	54	15
					L MFG	−51	36	30
					L MFG	−45	39	24
	R thalamus	461	5.26	>0.001	R thalamus	9	−15	9
					R thalamus	21	−30	6
					R thalamus	6	−27	−6
	R insula	121	5.07	0.015	R insula	30	21	0

Clusters significant at 0.05 after FWE correction. Up to 3 largest peaks listed per cluster L, left; R, right; STG, superior temporal gyrus; PHG, parahippocampal gyrus; MTG, middle temporal gyrus; ITG, inferior temporal gyrus; MFL, medial frontal lobe; OFC, orbitofrontal cortex.

### Association versus Conceptual Similarity

**Figure 2.**
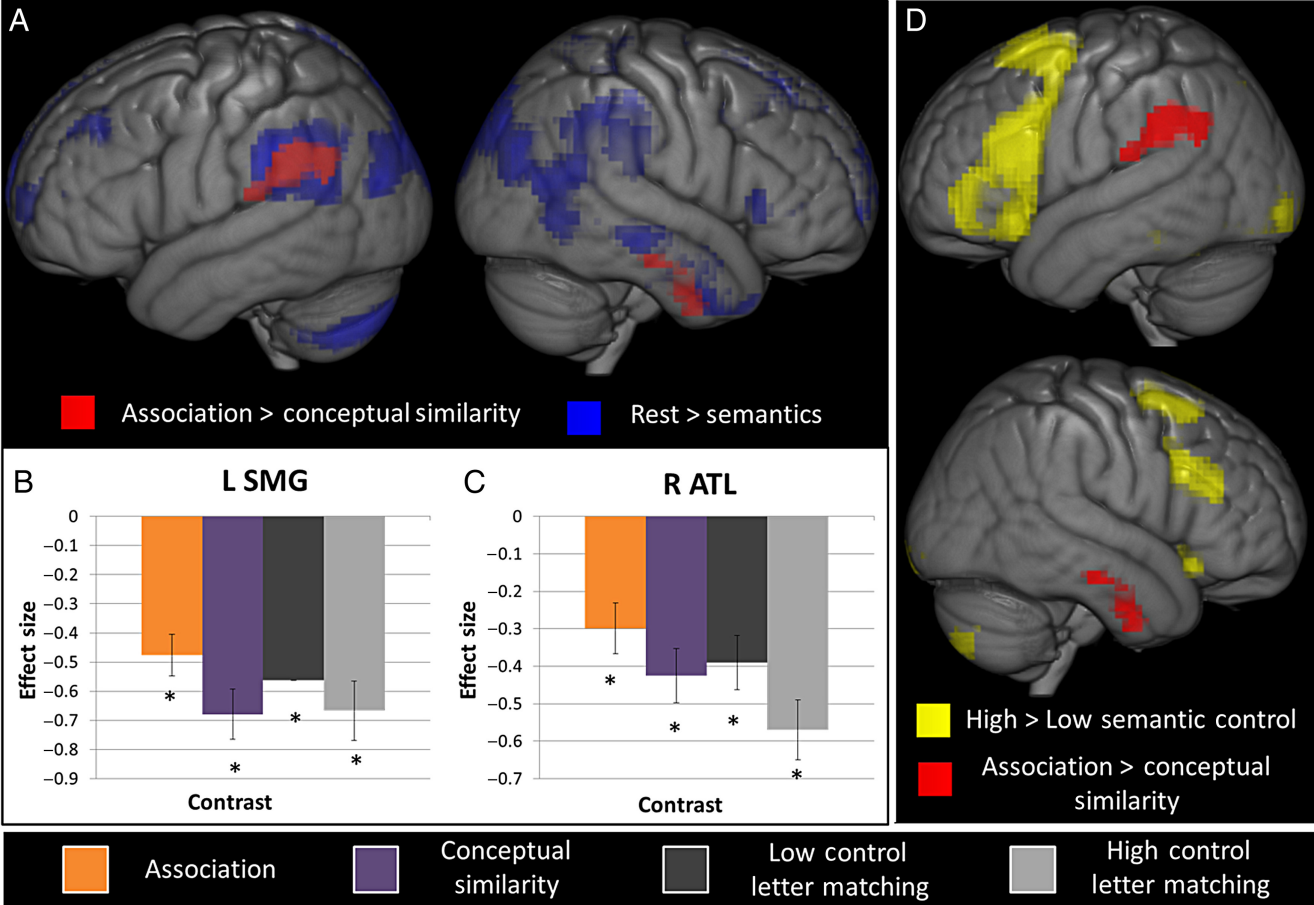
Assessment of the areas found for the association > conceptual similarity contrast without RT included in the model. (*A*) Areas with significantly greater activation for the contrast association > similarity (red) shown over the significant regions for the contrast rest > semantic (blue). (*B*) Effect sizes for a 10-mm spherical ROI centered around the peak of activity in the left supramarginal gyrus within the contrast association > conceptual similarity for the conditions associative (orange), conceptual similarity (purple), low control letter matching (dark gray) and high control letter matching (light gray) over rest. (*C*) Effect sizes for a 10-mm spherical ROI centered around the peak of activity in the right anterior temporal lobe within the contrast association > conceptual similarity for the conditions associative (orange), similarity (purple), low control letter matching (dark gray) and high control letter matching (light gray) over rest. Asterisks denote significant contrasts at *P* < 0.05 after application of a Bonferroni correction for multiple comparisons. Both ROIs show deactivation from rest for both forms of semantic relationship and the letter matching task (easy vs. hard conditions). Thus, the association > conceptual similarity contrast is due to differences in deactivation. No differences are significant if RT is included in the model (see text). (*D*) Significant activation for the contrast high semantic control > low semantic control (yellow) is overlaid on the association > conceptual similarity contrast (red). These regions did not overlap. Voxels significant at 0.001 with an FWE correction at the cluster level with a critical cluster level of 0.05.

**Figure 3.**
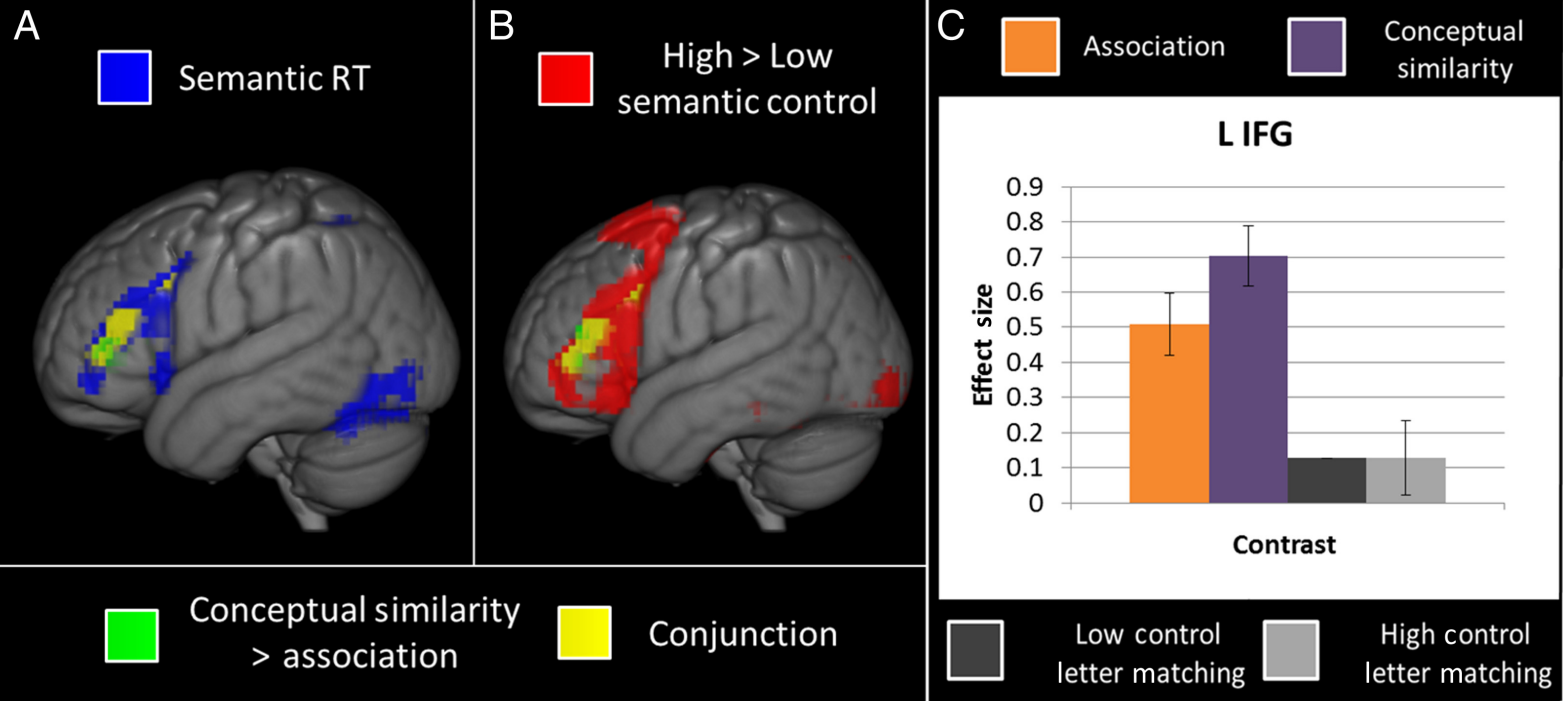
(*A*) A conjunction analysis of the contrast conceptual similarity > association (green) and the areas responding more to trials with long reaction times in the semantic task (blue). Areas of conjunction are shown in yellow. A high level of conjunction can be identified. (*B*) A conjunction analysis of the contrasts conceptual similarity > association (green) and high semantic control > low semantic control (red). Areas of conjunction are shown in yellow. A high level of conjunction is present. Voxels included in the conjunction analyses were significant at 0.001 with an FWE correction at the cluster level with a critical cluster level of 0.05. The differences between association and conceptual similarity may be explained by the level of difficulty of a general semantic process. No differences between association and conceptual similarity are significant if RT is included in the model. (*C*) Effect sizes for a 10-mm spherical ROI centered around the peak of activity in the left inferior frontal gyrus within the contrast conceptual similarity > association for the conditions associative (orange), conceptual similarity (purple), low control letter matching (dark gray), and high control letter matching (light gray) over rest. Asterisks denote significant contrasts (*P* < 0.05) after application of a Bonferroni correction for multiple comparisons.

Semantic trials were split into those based on associative relationships versus conceptual similarity to assess to what extent they share neuronal bases. Significant activation maps for each type of judgment over the letter matching (baseline) task are shown in Figure 1. The principal finding is clear—both judgment types resulted in a large common area of activity. In order to assess whether any areas responded differentially, a direct comparison of the 2 types of semantic relationship was performed, highlighting small differences generally outside of the large shared cluster for semantic judgments (see Figs 2 and 3as well as Table 3). Greater activation was found for associative > conceptual similarity in left supramarginal gyrus extending inferiorly to include STG and posteriorly to include the angular gyrus (see Fig. 2) and in the right ITG extending to MTG. The opposite (conceptual similarity > associative) contrast revealed a difference in the left inferior frontal gyrus, extending into precentral gyrus, and in a cluster comprising bilateral supplementary motor area, left superior medial frontal cortex, and right mid cingulum (see Fig. 3).

Further analyses were conducted at the whole-brain level to assess whether these differences could be explained in terms of 2 key performance factors: the required level of semantic control and generic difficulty (as measured by RT). Prefrontal regions (and other areas) have been implicated in the executive regulation of semantic processing (Thompson-Schill et al. 1997; Wagner et al. 2001; Badre and Wagner 2003; Badre et al. 2005; Noonan et al. 2013) and thus exhibit heightened activation for more difficult semantic judgments or tasks. In line with these many previous studies, the contrast of high > low control semantic judgments revealed large areas of the frontal and occipital lobes as well as the inferior and superior parietal lobes and fusiform gyrus extending to inferior temporal and parahippocampal gyri (see Fig. 2*D* and Table 4) . Conjunction analyses of the conceptual similarity > association contrast with this semantic control manipulation (see Fig. 3*B*) and with the RT in the semantic trials (see Fig. 3*A*) showed large areas of conjunction. This indicates that the significantly greater activity for conceptual similarity > associative judgments observed in inferior frontal and supplementary motor areas can be explained in terms of the greater executive demands of these more difficult semantic judgments. No overlap was found between semantic control regions and those revealed by the associative > conceptual similarity contrast (see Fig. 2*D*).

**Table 3:** Significant activation clusters for the direct comparison of association and conceptual similarity

Contrast	Region of activation	Cluster extent (voxels)	Max *z* value	*P* value (FWE corrected)	Peak MNI coordinate		
					*X*	*Y*	*Z*
Association > conceptual similarity	R inferior temporal gyrus	131	4.17	0.038	54	−9	−27
Association > conceptual similarity	L supramarginal and angular gyrus	161	3.76	0.018	−63	−45	36
Conceptual similarity > association	L inferior frontal gyrus	728	5.38	>0.001	−42	30	6
Conceptual similarity > association	L supplementary motor area	150	3.92	0.024	−6	12	54

Clusters significant at 0.05 after FWE correction. Largest peak listed per cluster.

L, left; R, right.

**Table 4:** Significant activation clusters for the semantic control manipulation (high control conceptual similarity > low control conceptual similarity)

Region of activation	Cluster extent (voxels)	Max *z* value	*P* value (FWE corrected)	Peak MNI coordinate		
				*X*	*Y*	*Z*
L inferior frontal gyrus	2386	6	>0.001	−51	15	27
L calcarine sulcus	519	4.93	>0.001	−9	−96	−9
R inferior frontal gyrus	373	4.9	>0.001	48	18	27
R mid frontal gyrus	253	4.76	0.001	36	21	54
L fusiform gyrus	210	4.63	0.004	−39	−21	−24
R inferior orbitofrontal cortex	153	4.63	0.016	30	24	−6
R calcarine sulcus	168	4.11	0.011	18	−93	−3
L inferior parietal cortex	194	4.11	0.006	−30	−69	45

Clusters significant at 0.05 after FWE correction. Largest peak listed per cluster.

L, left; R, right.

Next, as the associative and conceptual similarity conditions differed in average reaction time (see above), an analysis was run to assess which areas differ according to semantic task RT (i.e., task difficulty) versus which regions differed by condition regardless of RT (indicating a true effect of the type of semantic relationship). Figure 3 shows conjunction analyses of the areas where activation is correlated positively with RT (see Fig. 3A) and the areas found to have higher activation for conceptual similarity judgments (the condition with the longer average RT, see Fig. 3*B*; see Behavioral Results and Supplementary Fig. 2 for the coordinates of peak activation). The results of these contrasts overlapped within the IFG, supporting the idea that conceptual similarity judgments activated this area to a greater extent simply because they were more demanding. The contrast of conceptual similarity > association with RT included in the same model found no significant differences. Therefore, these regions were not activated more for conceptual similarity per se, but rather for trials of any relationship type requiring more effortful semantic processing.

Finally, we considered areas that were deactivated by the semantic task, relative to rest. Both the left supramarginal gyrus and right ATL clusters identified in the associative > conceptual similarity contrast overlapped with a broader set of regions which showed significant deactivation from rest regardless of task (see Fig. 2*A* and Supplementary Table 1). As shown in Figure 2*B*, the difference between the associative and conceptual similarity-based trials in these areas reflected a differential deactivation. Various previous studies, across different cognitive domains, have demonstrated that the deactivation, commonly observed in ventral parietal cortex (a part of the default-mode network), is anticorrelated with task difficulty (Fox et al. 2005; Buckner et al. 2008; Harrison et al. 2011; Gilbert et al. 2012; Humphreys and Lambon Ralph 2015). Indeed, the differential deactivation for associative versus conceptual trials observed in ventral parietal cortex was no longer significant when reaction time was included as a parametric regressor. This means that deactivation of these regions does not differ by relationship type but rather reflects the difficulty of general semantic processing. In fact, no differences were found between the association and conceptual similarity trials anywhere in the brain when semantic RT was included in the model. Differential activation in a number of regions was found to relate to indices of general semantic difficulty but no differences were shown to relate to relationship type, per se.

### Region-of-Interest Analyses

**Figure 4.**
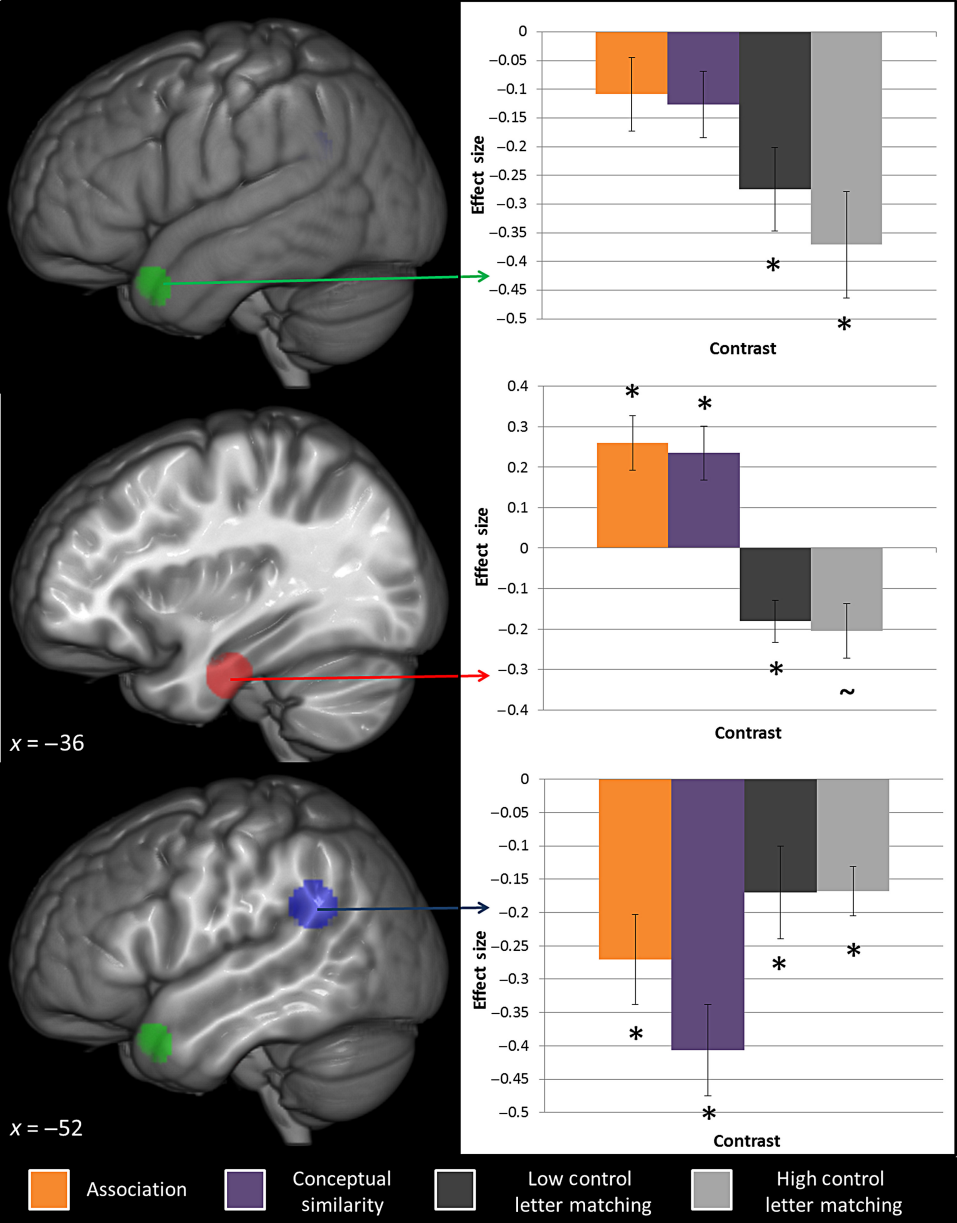
Location and effect sizes of the 3 ROIs. Schwartz et al.'s (2011) aSTS region is shown in green (MNI coordinates = −53 18 −30) with the ventral ATL ROI from Binney et al. (2010) in red (MNI coordinates = −36 −15 −30). Schwartz et al.'s (2011) TPJ ROI is displayed in blue (MNI coordinates = −52 −49 27). The effect sizes of each condition against rest are displayed for each ROI for the conditions associative (red), similarity (green), low control letter matching (light blue), and high control letter matching (dark blue) over rest. Asterisks denote significant contrasts after application of a Bonferroni correction for multiple comparisons (*P* < 0.05). Tilde denotes a trend toward significance (*P* < 0.1).

Region-of-interest analyses were conducted to test the dual-hub model's predictions that ATL is involved specifically in coding conceptual similarity-based relationships and TPC in associative relationships. One ATL ROI located within anterior STS was taken from Schwartz et al. (2011), who had identified this region as a potential representational hub for conceptual similarity (with respect to speech production). Another, from the ventral ATL, was taken from Binney et al.'s (2010) distortion-corrected fMRI study of synonym judgments, which has been proposed as the centerpoint of a graded transmodal semantic hub (Lambon Ralph et al. 2010; Lambon Ralph 2014). This ventral ATL area has been found in multiple imaging studies across tasks and modalities (Marinkovic et al. 2003; Sharp et al. 2004; Spitsyna et al. 2006; Visser and Lambon Ralph 2011; Visser et al. 2012) and is an area of maximal atrophy and hypometabolism in semantic dementia, which directly correlates with their degree of semantic impairment (Galton et al. 2001; Butler et al. 2009; Mion et al. 2010). The coordinates of the TPJ ROI was taken from Schwartz et al. (2011) in order to assess the claim that this region reflects the site of a hub for associative semantics. The TPC peak was located at the junction of Brodmann areas 21, 22, 39, 40, 41, 42, and 48 (see Fig. 4).

The results of the ROI analyses for each contrast are listed in Table 5 and summarized in Figure 4. Neither ATL ROI showed a significant difference between association and conceptual similarity judgments (see Table 5), with the ventral ATL ROI showing the strongest activations for both semantic conditions over the letter matching baseline. The superior ATL ROI also showed significantly greater yet equivalent activation for the semantic judgments over the active baseline (which was deactivated with respect to rest). Indeed, these results underline previous observations that ATL semantic activations are much more likely to be detected when an active baseline is used (see Visser et al. 2010). In line with the whole-brain analyses, these ROI findings underline the conclusion that the ATL is implicated in general semantic representation regardless of relationship type (see Fig. 4). In contrast to the ATL ROIs, all conditions showed significant deactivation from rest within the TPJ ROI. Neither conceptual similarity nor association conditions were significantly more de-activated than the letter matching task, or each other. Deactivation did not relate to reaction time or semantic control. Thus, counter to the dual-hub hypothesis, this study found no evidence in favor of this region supporting associative semantics.

**Table 5:** Independent ROI analyses

ROI	Contrast	Effect size	*T* value	Bonferroni-corrected *P* value
ATL (Schwartz)	Association > rest	−0.11	−1.69	0.937
	Conceptual Similarity > rest	−0.13	−2.19	0.347
	Low control letter matching > rest	−0.27	−3.77	<0.05
	High control letter matching > rest	−0.37	−4.00	<0.05
	Association > letter matching	0.43	3.90	<0.05
	Conceptual similarity > letter matching	0.39	3.50	<0.05
	Association > conceptual similarity	0.02	0.47	1
	High > low semantic control	0.00	0.01	1
	High > low nonsemantic control	−0.10	−1.84	0.703
ATL (Binney)	Association > rest	0.26	3.87	<0.05
	Conceptual similarity > rest	0.23	3.53	<0.05
	Low control letter matching > rest	−0.18	−3.47	<0.05
	High control letter matching > rest	−0.20	−3.02	0.055
	Association > letter matching	0.90	9.07	<0.001
	Conceptual similarity > letter matching	0.85	8.69	<0.001
	Association > conceptual similarity	0.03	1.05	1
	High > low semantic control	0.06	4.85	<0.001
	High > low nonsemantic control	−0.02	−0.47	1
L TPC (Schwartz)	Association > rest	−0.27	−4.02	<0.05
	Conceptual similarity > rest	−0.41	−5.93	<0.001
	Low control letter matching > rest	−0.28	−4.03	<0.05
	High control letter Matching > rest	−0.39	−4.81	<0.001
	Association > letter matching	0.13	1.29	1
	Conceptual similarity > letter matching	−0.15	−1.51	1
	Association > conceptual similarity	0.14	2.75	0.104
	High > low semantic control	0.00	0.12	1
	High > low nonsemantic control	−0.11	−1.98	0.541

## Discussion

The clear, principal finding from this study was that semantic judgments based on either associative relationships or conceptual similarity engaged the same neural network, including bilateral ATL, posterior temporal regions and left IFG. These areas fit well with previous neuroimaging and neuropsychological findings regarding their roles in general, multimodal semantic cognition (Thompson-Schill et al. 1999; Jefferies and Lambon Ralph 2006; Vigneau et al. 2006; Patterson et al. 2007; Binder et al. 2009; Binney et al. 2010; Binder and Desai 2011; Visser et al. 2012; Noonan et al. 2013) and suggest that both forms of semantic knowledge arise from a single network (see below).

The 3 issues presented in the Introduction section were elucidated. The selective differences between the ATL and TPC predicted by a strong version of the dual-hub model were not identified in whole-brain or ROI analyses. We found little evidence for differential activation in the regions hypothesized, under the dual-hub proposal, to represent associative and conceptual similarity separately. The expectation from single hub-and-spoke model of significant positive activation of the ATL for both relationship types was met. The ATL was strongly and significantly activated by semantic judgments regardless of relationship type, consistent with both its proposed role as a graded, transmodal, pan-category representational hub (Patterson et al. 2007; Lambon Ralph et al. 2010; Lambon Ralph 2014), and the poor performance on both types of judgment exhibited by patients with semantic dementia (in the context of ATL-centered atrophy: Jefferies and Lambon Ralph 2006; Butler et al. 2009; Hoffman, Jones et al. 2013).

The role of the TPC was less clear as both types of semantic relationship showed deactivation from rest, as did the letter matching baseline task and no significant differences were found between association and conceptual similarity. This area is part of a wider region associated with deactivation from rest, including ventral parietal cortex, that is, the default-mode network (e.g., Buckner et al. 2008). Although no differences between the 2 conditions were found in the TPC, the effect of general semantic control and difficulty was found to be critical for understanding the role of regions outside the ROIs. Beyond these 2 regions, the only areas to be identified as more active for conceptual similarity than associative semantic judgments were the inferior prefrontal cortex and supplementary motor area. This result reflected differential semantic difficulty (highlighted by the overlap of conceptual similarity > associative and hard > easy semantic judgment contrasts) and is consistent with inferior prefrontal cortex's role on controlled semantic processing as demonstrated by previous neuroimaging, neuropsychological and TMS studies (Thompson-Schill et al. 1997; Wagner et al. 2001; Badre and Wagner 2003; Badre et al. 2005; Jefferies and Lambon Ralph 2006; Hoffman et al. 2010; Noonan et al. 2013). Indeed, after accounting for the differences in reaction time between the 2 conditions, no areas were found to exhibit differences between the 2 types of semantic judgment. The importance of measures of general semantic difficulty was highlighted as differences appearing to relate to relationship type were shown to relate to semantic difficulty alone. This included differences in both positive activation and in deactivation.

By keeping the task instructions the same and giving no explicit direction to use different sub-types of knowledge for the 2 conditions, we assessed whether there was an automatic, neural distinction between association and conceptual similarity. It is possible that explicit task instructions could differentially enhance activation of one subtype of conceptual knowledge and drive greater variation in the activation of distinct neural regions (Wisniewski and Bassok 1999). This could be explored in future studies. However, the negative side of this approach would be that any resultant differences might reflect metacognitive processes rather than the type of knowledge.

### Relationship to Previous Neuropsychological Findings

The results correspond well with neuropsychological data. Both semantic aphasia and semantic dementia patients show impairment on explicit tests of semantic associations and conceptually similar items, as well as impaired feature knowledge (Bozeat et al. 2000; Jefferies and Lambon Ralph 2006; Hoffman, Jones et al. 2013). This is because both groups have damage to the general semantic network found to code associations and conceptual similarities; in semantic dementia to the ATL and in semantic aphasia to pMTG, ventral parietal cortex or IFG (Jefferies and Lambon Ralph 2006; Patterson et al. 2007). Previously, Goldstein (1936, 1948) considered semantic aphasic patients to be overly sensitive to associative relationships, with little influence of conceptual similarity. However, these clinical observations were not controlled to the same extent as this experiment. Indeed, these conclusions may arise, in part, from the underestimation of the importance of associative thinking in healthy adults, identified more recently (Lin and Murphy 2001).

Despite the consistency between this experiment and existing neuropsychological data, the same harmony does not hold if rates of different types of speech errors are measured. If association and conceptual similarity rely on the same multimodal semantic network, why are different semantic error types linked to different regions after brain damage? Semantic aphasic patients make a mixture of associative, categorical and superordinate semantic errors, whereas semantic dementia patients rarely, if ever, produce associative semantic errors (Jefferies and Lambon Ralph 2006). Although generating fewer errors overall, neurologically intact participants generate the same ratio of semantic error types as that observed in a large group of patients with post-stroke aphasia (Schwartz et al. 2011). The naming errors in SD are consistent with the progressive collapse and degradation of the underpinning semantic representations (Lambon Ralph et al. 2001). The characteristic of this semantic impairment is that it is increasingly difficult for the semantic system 1) to separate conceptually similar items (leading to category and superordinate errors) and 2) to generate specific information linked to each concept, including its name (the most common error type in SD is an omission error), specific features and associations (Warrington 1975; Lambon Ralph et al. 2001). The inability to generate detailed information about each concept will mean that associative naming errors are very unlikely. Indeed, Jefferies and Lambon Ralph (2006) noted that the presence of conceptually specific associative errors in SA (e.g., squirrel → “nuts”) implies a very good underlying semantic database.

These factors probably explain, at least in part, the innovative voxel-based lesion-symptom mapping (VLSM) results reported by Schwartz et al. As lesions encroach upon ATL regions, category-related errors will tend to increase and associative errors decrease (as per SD patients). The second effect to account for in the Schwartz et al. study is the “relative” increase in associative over categorical errors linked to TPC lesions. Perhaps the most obvious possibility follows from the fact that speech production is complex and involves multiple stages (Dell and Reich 1981). Associative errors may arise from a nonsemantic stage linked to TPC or a nearby area. For instance, the angular gyrus has been shown to activate for sentence-level and syntactical processing (Petersson et al. 2012; Zhu et al. 2012). It is entirely possible that these mechanisms may partially activate lexically associated words (a natural outcome of their role in connected-speech and sentence construction) and, under damage or poor control, these alternatives are incorrectly produced by the patients during picture naming tasks. A second possibility is statistical. Given that Schwartz et al. (2011) reported “partial” correlations (categorical|associative vs. associative|categorical), it is possible that the presence of patients with ATL lesions and less associative errors within the entire dataset will automatically generate a mirror-image partial correlation for the remaining patients with non-ATL MCA lesions. This is consistent with the fact that, in the patient data overall, the ratio of different semantic error types was the same that observed in neurologically intact participants. If this explanation is correct then there is, in effect, only a single dissociation present in those results (ATL lesions decreasing the rate of associative errors). If, however, there was an absolute increase in associative errors in the TPC subgroup (i.e., significantly more than that observed in general aphasic and control groups, overall) then an alternative explanation is required.

### How Could Association and Conceptual Similarity Arise Out of One Representational System?

The primary result of this study was that processing of semantic associations and conceptual similarity rely upon the same semantic neural network. What does this imply for theories of semantic representation? First and foremost, it would seem to suggest that these 2 important forms of semantic knowledge are coded within a single neurocomputational system. Below, we consider how this might be achieved within a neuroanatomically inspired, computationally implemented framework such as the hub-and-spoke model (Rogers et al. 2004; Patterson et al. 2007; Lambon Ralph et al. 2010; Lambon Ralph 2014).

The key ideas are as follows. Concepts are built from, and reflect the characteristics of, our multimodal experiences which are acquired, typically, over a long period of time. Registration of the information arising in each input/output modality (“engrams” in the classical neurological accounts of conceptualization; Eggert 1977) is achieved within secondary association cortices (the spokes within the hub-and-spoke framework). According to one implemented computational model (Rogers et al. 2004), these different sources of information are drawn together through interaction with a transmodally connected representational hub (centered on the ventrolateral ATL; Binney et al. 2012) which integrates over time, contexts and modalities to extract generalizable, coherent conceptual representations and computes the many nonlinear relationships between each concept and its linked elements or “features” of knowledge (Lambon Ralph et al. 2010; Lambon Ralph 2014). It is the co-occurrence of features identified by the hub that gives rise to semantic structure and conceptual similarity in computational models, hierarchical cluster analysis and feature databases (Rogers et al. 2004; Dilkina and Lambon Ralph 2013). Graded conceptual similarity is an emergent property of this computational framework and reflects these deeper statistical structures present in our multimodal experience (Rogers et al. 2004; Lambon Ralph et al. 2010). Indeed, the model captured not only hierarchical, taxonomic-like structure, where it exists (e.g., within natural categories) but also strong and weak similarities among other types of (nontaxonomic) concept.

Although not considered explicitly in the original computational exploration, it is possible that the same framework would code associations between concepts in the same way as the link between any concept and its “features”. Indeed, it is possible that “features” and “associations” are one and the same thing—i.e., the smorgasbord of information that is linked to a concept. Specifically, the model learns to map between a concept and all of its associated/linked information (as described in the Introduction for croissant). The verbal and nonverbal “features” of croissants (e.g., the name “croissant,” “crescent shaped,” “edible,” etc.) are simply elements of experience that reliably co-occur in time and context, and therefore coalesce to form an integrated concept of the object. From this perspective, “associations” (e.g., <coffee>) can be thought of as additional elements of experience that are also often present and thus become integrated into the concept. In other words, there is no strong distinction between an item's “associations” and its “features.” They are all simply aspects of the environment that are experienced together when the item is encountered.

The information that is linked to each concept (whether “features” or “associations”) varies along at least 3 different dimensions: 1) in which sensory-verbal modalities it is experienced; 2) the range of concepts to which each piece of information/feature is linked (i.e., shared vs. distinctive features: Garrard et al. 2005; Tyler et al. 2013); and 3) its experiential frequency (i.e., how often each piece of information is experienced alongside the concept—e.g., “buttery taste” and croissant are very commonly paired but “chocolate filling” is a less frequent feature). Both “features” and “associates” can vary in their specificity (applicable to individual or collections of concept) and can be extracted from verbal or nonverbal experience. Even a distinction between internal (e.g., parts of the object) versus external (i.e., present in the environment outside of that object; as described in Lin and Murphy 2001) information does not necessarily distinguish between “features” and “associations” given that, like associations, many “features” are external to the object (e.g., “buttery smell,” “flaky texture,” etc.). We should note here that “associates” have the key characteristic of co-occurring in time or place, verbally (e.g., lexical associates “French croissants”) or nonverbally (e.g., seeing croissants and coffee next to each other). Our working hypothesis does not reject this fact but rather observes that this is true to varying degrees (i.e., experiential frequency) for all information/“features” linked with a concept. Second, if different cognitive and neural systems code conceptual similarity versus “association” structures then a potential homunculus problem arises in terms of which subsystem should code the information (e.g., are “warmed in the oven,” “made from a yeast dough,” features or associations?). How do associates and features relate to the task used here? In conceptual similarity trials, presentation of the probe and target concepts activates many overlapping features. These shared features allow the participant to choose the target as related to the probe. As features are learnt throughout life, the amount of overlap between sets of concepts forms a structure reflecting conceptual similarity, thus allowing conceptual similarity to emerge from the co-activation of sets of features. In the association trials, the same sets of co-activated features become engaged in the same way but here the sum difference is not important. Instead, some of these activated features are generally termed “associates” and identification of this link allows the participant to choose the target item. In summary, according to this hypothesis, each concept is linked or associated with a range of verbal and nonverbal experiential information, and conceptual similarity reflects the deeper statistical structure extracted across these concept-to-associations/features structures.

Finally, we note that associations could be considered to be one of a number of kinds of representation responsible for coding sequences and time-based information (e.g., schemas, syntax, etc.). Although semantic associations were not found to rely on a distinct cortical network, this does not mean that there are no systems for coding temporal or spatial statistical structures. Rather, it seems likely that these structures are orthogonal to semantically related representations and are coded in different neural regions, such as the ventral parietal cortex, which has been shown to be involved in processing syntax, numbers, and space (e.g., frontoparietal “dorsal” vs. “ventral” temporal lobe pathways: Walsh 2003; Ueno et al. 2011; Petersson et al. 2012; Zhu et al. 2012; Bornkessel-Schlesewsky and Schlesewsky 2013).

## Conclusions and Future Directions

When general semantic difficulty is accounted for, association and conceptual similarity rely on the same cortical network responsible for semantic cognition including ATL, posterior temporal, and inferior frontal cortex. In order to gain further insights about how association and conceptual similarity may relate, future computational models of semantic cognition should address 2 challenges. If there is indeed no strong distinction between features and associations, models should be able to demonstrate both how associations and other features are linked to each concept, as well as how the deeper, graded conceptual similarities emerge out of a unified framework as suggested by the current neuroimaging and neuropsychological data from semantic dementia and semantic aphasia. In addition, successful models should be able to show how the differential distributions of aphasia naming errors arise from this unified framework.

## Supplementary Material

Supplementary material can be found at: http://www.cercor.oxfordjournals.org.

## Funding

This work was supported by an MRC program grant to M.A.L.R. (grant number MR/J004146/1). R.L.J. was supported by an EPSRC-funded studentship. Funding to pay the Open Access publication charges for this article was provided by an RCUK block grant to the University of Manchester.

## Supplementary Material

Supplementary Data
