# Supplementary material for: The Nature and Neural Correlates of Semantic Association versus Conceptual Similarity
Source: Cereb Cortex. 2015 Jan 30;25(11):4319–33. doi: 10.1093/cercor/bhv003 (PMC4816784; doi:10.1093/cercor/bhv003)
Supplement: Supplementary Data [file supp_25_11_4319__index.html]

The Nature and Neural Correlates of Semantic Association versus Conceptual Similarity — The Nature and Neural Correlates of Semantic Association versus Conceptual Similarity — Supplementary Data 

# The Nature and Neural Correlates of Semantic Association versus Conceptual Similarity

## Supplementary Data

Supplementary Data

**Files in this Data Supplement:**

- Supplementary Data - Docx file
