# Supplementary material for: The Nature and Neural Correlates of Semantic Association versus Conceptual Similarity
Source: Cereb Cortex. 2015 Jan 30;25(11):4319–33. doi: 10.1093/cercor/bhv003 (PMC4816784; doi:10.1093/cercor/bhv003)
Supplement: Supplementary Data [file supp_bhv003_bhv003supp.docx]

**Supplementary Material**

S1. Significant activation clusters for the contrast rest > semantic.

| Region of Activation | Cluster extent (voxels) | Max z value | P value (FWE corrected) | Peak regions | Peak MNI Coordinate | | |
| --- | --- | --- | --- | --- | --- | --- | --- |
|  |  |  |  |  | X | Y | Z |
| Bilateral occipital, parietal and frontal cortex, R temporal lobe | 12806 | 6.63 | >.001 | L mid cingulate gyrus | -12 | -39 | 39 |
|  |  |  |  | L precuneus | -12 | -48 | 51 |
|  |  |  |  | R MTG | 42 | -72 | 21 |
| L FG & lingual gyrus | 114 | 5.94 | .032 | L FG | -27 | -48 | -9 |
| L Cerebellum | 547 | 5.70 | >.001 | L Cerebellum | -24 | -78 | -42 |
|  |  |  |  | L Cerebellum | -33 | -78 | -39 |
|  |  |  |  | L Cerebellum | -48 | -63 | -45 |
| R FG & lingual gyrus | 139 | 5.63 | .015 | R FG | 30 | -51 | -9 |
| L ventral parietal cortex | 573 | 5.44 | >.001 | L SMG | -63 | -33 | 36 |
|  |  |  |  | L AG | -63 | -60 | 30 |
|  |  |  |  | L AG | -51 | -69 | 45 |

Clusters significant at .05 after FWE correction. Up to 3 largest peaks listed per cluster. L = left. R = right. MTG = middle temporal gyrus. FG = fusiform gyrus. SMG = supramarginal gyrus. AG = angular gyrus.

S2. Significant activation clusters for the reaction time modulator.

| Region of Activation | Cluster extent (voxels) | Max z value | P value (FWE corrected) | Peak MNI Coordinate | | |
| --- | --- | --- | --- | --- | --- | --- |
|  |  |  |  | X | Y | Z |
| L IFG | 1201 | 5.46 | >.001 | -48 | 30 | 18 |
| R IFG | 1136 | 5.42 | >.001 | 48 | 33 | 18 |
| R mid occipital lobe | 425 | 5.4 | >.001 | 33 | -63 | 30 |
| L ITG | 4032 | 5.4 | >.001 | -48 | -57 | -15 |
| R mid cingulum | 512 | 4.8 | >.001 | 3 | 21 | 42 |

Clusters significant at .05 after FWE correction. Largest peak listed per cluster. L = left. R = right. MTG = middle temporal gyrus. IFG = inferior frontal gyrus. ITG = inferior temporal gyrus.

S3. Stimuli presented in the semantic judgement task.

| Probe | Associated | | Similar | |
| --- | --- | --- | --- | --- |
|  | Target | Foil | Target | Foil |
| bird | egg | tent | lamb | blacksmith |
| shop | basket | spoon | market | bottle |
| hoover | dirt | tomb | mower | synagogue |
| vase | tulip | elephant | bucket | platform |
| apple | orchard | map | lime | lecturer |
| exam | lecturer | eagle | programme | road |
| graffiti | wall | market | poster | saddle |
| finger | glove | dress | claw | referee |
| prisoner | dungeon | medal | pupil | pony |
| truck | road | college | gondola | duvet |
| yacht | bay | oven | automobile | basket |
| suit | tailor | beaver | robe | organ |
| trail | forest | rattle | street | athletics |
| dolphin | ocean | tale | pig | officer |
| badge | officer | student | medal | baggage |
| warehouse | pallet | gun | mansion | pond |
| pot | plant | chicken | pail | costume |
| shovel | hole | mosque | spoon | meal |
| surgeon | scalpel | pail | butcher | camel |
| rat | maze | monastery | otter | bride |
| flask | gin | slipper | goblet | maze |
| leopard | zoo | auditorium | fox | warrior |
| hen | cage | robe | robin | baby |
| panda | bamboo | nettle | mouse | author |
| tumbler | whiskey | trolley | chalice | scalpel |
| ferry | island | till | canoe | mug |
| pike | river | bill | shark | toe |
| armour | blade | submarine | dress | wall |
| python | jungle | stout | worm | detective |
| bell | tower | axe | rattle | gin |
| lorry | highway | furnace | limousine | trench |
| hair | brush | street | fur | vicar |
| portrait | gallery | juice | report | orchard |
| warrant | detective | ferret | bill | zoo |
| grass | rake | flute | lily | dentist |
| car | mechanic | pupil | wagon | barn |
| boot | toe | vine | slipper | burrow |
| jeweller | necklace | programme | student | plant |
| jail | robber | lobster | tomb | honey |
| fort | infantry | sack | bungalow | ink |
| abbey | monk | policeman | lodge | egg |
| squirrel | woodland | tank | ferret | deity |
| grill | sausage | chalice | furnace | jungle |
| whistle | referee | butcher | flute | tower |
| teeth | dentist | lime | beak | bamboo |
| bee | honey | automobile | eagle | choir |
| helmet | bike | limousine | crown | restaurant |
| muzzle | dog | brain | mask | ocean |
| seaweed | sand | park | nettle | tailor |
| lemonade | bottle | school | liquor | marsh |
| cocktail | bar | meadow | stout | harbour |
| tractor | hay | coat | tank | necklace |
| waitress | restaurant | coach | nurse | flower |
| desert | camel | robin | meadow | rifle |
| cart | pony | otter | jeep | cage |
| lager | glass | mower | juice | bay |
| reed | marsh | mansion | vine | mechanic |
| menu | meal | concrete | map | web |
| snake | venom | cape | lobster | monk |
| bayonet | trench | report | axe | bike |
| spider | web | canoe | lice | pastor |
| printer | ink | pasture | till | woodland |
| veil | bride | fox | cape | gallery |
| boat | harbour | essay | coach | dungeon |
| cloak | friar | mouse | coat | sand |
| millstone | corn | lice | concrete | infantry |
| forge | blacksmith | shark | oven | island |
| theatre | costume | jeep | saloon | dirt |
| cat | vet | lily | mole | friar |
| hymn | organ | photo | tale | forest |
| bedroom | duvet | gondola | dormitory | sausage |
| farm | sheep | bear | park | pallet |
| soldier | rifle | bucket | policeman | sheep |
| pulpit | pastor | pig | podium | venom |
| airport | baggage | bungalow | school | milk |
| stadium | athletics | poster | auditorium | hay |
| hotel | guest | fur | monastery | river |
| monarch | throne | liquor | president | corn |
| temple | deity | haddock | college | glass |
| tea | mug | mask | wine | blade |
| garden | flower | beak | pasture | throne |
| train | platform | lodge | submarine | brush |
| book | author | nurse | photo | highway |
| church | vicar | lamb | mosque | rake |
| pram | baby | bishop | trolley | hole |
| cow | milk | goblet | bear | robber |
| bible | god | president | essay | whiskey |
| cathedral | choir | claw | tent | beer |
| womb | midwife | mole | brain | vet |
| sword | warrior | worm | gun | bar |
| rabbit | burrow | podium | beaver | midwife |
| rabbi | synagogue | wine | bishop | dog |
| goldfish | pond | saloon | haddock | tulip |
| owl | barn | wagon | chicken | god |
| horse | saddle | crown | elephant | guest |
| keg | beer | dormitory | sack | glove |

S4. Stimuli presented in the letter matching task.

| Probe | Target | Foil |  |
| --- | --- | --- | --- |
|  |  | Low control | High control |
| ##HΨz## | bqwcHΨz | ctkdLXQ | cHΨdLXQ |
| ##XΘm## | bXΘmPkt | gYBgTTN | gYBgTΘm |
| ##Bαj## | BαjgGdT | TCkDQXz | TCkDαjz |
| ##Rζl## | CGWWζlw | gXjBPxD | gXjζPxD |
| ##Hθm## | CHθmDZw | TYnbxyy | TYθmxyy |
| ##bΘp## | cKNΘpRw | NyMlmTw | NyMΘmTw |
| ##ZΣY## | CPΣYqFg | wdXpJSb | wdΣpJSb |
| ##CηI## | CηlQpjM | PHWxSQs | PηlxSQs |
| ##QΛL## | dQΛspDX | zpmhzYf | zpΛhzYf |
| ##SΠG## | DΠGzHXW | HzXJfbD | HzXΠfbD |
| ##cξt## | FNqFcξt | QdjMjMB | QdjcξMB |
| ##rΦM## | gBrΦMln | kzQGSCC | krΦGSCC |
| ##zχb## | gDcTzχb | nPXcnmP | nPXlzχP |
| ##XλP## | GfNjXλg | LtFcCdD | LtFcCλD |
| ##cπk## | HMcπkBX | PgdrYZl | PπkrYZl |
| ##jξs## | hQXjξsG | kFDfyCk | kFξsyCk |
| ##Rιx## | hQιxZSt | hqqGfwz | hqιGfwz |
| ##PΔY## | hrHΔYZL | KjhTDqR | KjhTΔqR |
| ##hγf## | hγfWkYM | lgCnxSM | lhγnxSM |
| ##Bξc## | JBξcHHF | nxqrkyg | nBξrkyg |
| ##Rδw## | JdRδwFf | XZsmKCF | XZsmKδw |
| ##hΔW## | jhΔRKtC | jNBzJjy | jNBΔJjy |
| ##GΦm## | JNmGΦmD | nDNKdLZ | GΦNKdLZ |
| ##KηN## | jpKηbbs | rytmfTR | rytηfTR |
| ##tτz## | JqrGtτz | bxpLYDY | bxpLYτz |
| ##xξm## | JRKtBξm | YZqpBBG | YZqpξBG |
| ##TπH## | JTπPGky | DsQdcKx | DsQdcπx |
| ##kΨF## | jxnBΨFX | gQyZlGZ | gQΨZlGZ |
| ##RΩw## | jYRΩwdg | bQhdBSk | bQhRΩSk |
| ##rΓD## | jZpHrΓD | smpQkwG | smpQkΓD |
| ##WηZ## | jηZtMmT | xppgTfj | xpηgTfj |
| ##Zθt## | jθtyPBc | DKQwBRn | DKQwBθn |
| ##Jτs## | JτYHdbf | NCBWMYg | NτBWMYg |
| ##bιF## | KbιFGPT | TZMxJqX | TZMxιFX |
| ##QΣX## | KhΣXhFT | pDNPJHd | pDNΣJHd |
| ##TβD## | KTβDzgJ | MwrbSgL | MwrβDgL |
| ##Bδg## | KzBδdPs | dXhZsTX | dXhδsTX |
| ##WΘJ## | kzmCWΘJ | xtKsXqp | xtΘJXqp |
| ##kυM## | kυMXynR | HXzdLns | HXυMLns |
| ##kψM## | kψMGrQW | fgDpRfF | fgDkψfF |
| ##yτW## | lfyτWcX | dxjBrGl | dxjτWGl |
| ##hΞw## | lhΞwMzt | GDlcksb | GΞwcksb |
| ##dΣM## | LNRdΣMW | KsxwfHf | KsxwΣMf |
| ##JΘX## | LySJΘXq | GzGMTKb | GJΘMTKb |
| ##lτb## | lτbfLcD | pkwjSXN | pkwτbXN |
| ##Hπl## | MDZπlxm | rbJwWRK | rbπwWRK |
| ##lΠB## | mlΠkTHY | JqydfWp | JqydΠWp |
| ##fμZ## | MNfμHtk | wbXTkMY | wbXTμMY |
| ##GΨW## | mQGΨWbn | YcjNxpH | YcjNGΨH |
| ##qμD## | mqμDjXZ | NKJXKGL | NKJqμGL |
| ##Qψy## | MrQψyNf | jLNcggl | jLQψggl |
| ##Sδp## | MxlKSδp | hNZcyhm | hNZcδpm |
| ##MΩB## | MΩxGslr | YCcrfnq | YCΩrfnq |
| ##MΔr## | nbcMΔrP | QFLFwdy | QFLΔrdy |
| ##KΣp## | NyzKΣpm | LWsbbZB | LWΣpbZB |
| ##zρy## | nzρpSfz | wXBHWFC | wXρHWFC |
| ##NΓR## | NΓRGBLH | cTSYfLd | cTSYfΓR |
| ##QδC## | nδCzjTY | WnNWrlF | WnNWδlF |
| ##qβg## | PBjDqβg | JWNkNrP | JβgkNrP |
| ##Dρw## | PkqQDρw | Lrgjbhc | LrDρbhc |
| ##Lθk## | PwDLθkp | HHFpFQY | HθkpFQY |
| ##xφT## | QLφTJrY | syzlhgH | sφzlhgH |
| ##gΛD## | QZcΛDCs | xjnzTWx | xΛnzTWx |
| ##JΞl## | RFbJΞlf | fRwKrPq | fRwΞlPq |
| ##kΓn## | rpSgkΓQ | sGpSbLG | sGΓSbLG |
| ##rβp## | rβpkGKd | YNChDmm | rβChDmm |
| ##zγK## | sCγKNJQ | qcPccRm | qcPcγRm |
| ##cΦH## | SfcΦPWx | BjbqKKl | BjΦqKKl |
| ##fλn## | sfλngkz | rspTlRd | fλpTlRd |
| ##pΨn## | SmJyΨnw | SYyCtHH | SYyCΨHH |
| ##FυQ## | SyFυQkh | MmyzZtK | MmyzυQK |
| ##Pεx## | SzRHεxB | nQtBkYw | nεtBkYw |
| ##sζP## | sζtTgNR | gjMxTwK | gζMxTwK |
| ##sφZ## | sφybtbW | rrHgJCB | rrHφJCB |
| ##bπy## | TcQXNπy | pShJfDK | pSπJfDK |
| ##jζK## | tjζKGgd | zLPXwDf | zLPjζDf |
| ##TΩR## | TΩRlLPc | yxYHKjb | yTΩHKjb |
| ##Tυh## | wgKTυQS | FrkWNLQ | FrkυNLQ |
| ##ZΠD## | WpftZΠS | SgFyHWN | SgFΠHWN |
| ##rΛL## | wScrΛxB | CnCnFQz | CnCnFΛz |
| ##hφG## | wsRhφGT | WtBqRCM | WtBhφCM |
| ##dμt## | WxZdμtY | BSHyGrW | BSHyGμt |
| ##yρf## | XDSBZρf | ndRLHjJ | ndRρHjJ |
| ##KζY## | xfKζYNR | SzxftZc | SzζYtZc |
| ##dωB## | xgdωFlr | RtJfWYk | RωJfWYk |
| ##Sγm## | xjTYSγm | ZKWyZzB | ZKWγmzB |
| ##tλf## | XlyFPλf | MWjDxZq | MWjDxλq |
| ##YΩW## | xXqlsΩW | thTgslL | thTgsΩL |
| ##GΠJ## | yGΠCZsk | tSFCpSs | tΠFCpSs |
| ##dψw## | YjgψwCB | GZtFMlS | GZtψMlS |
| ##Cμj## | yKtCμjh | bBXlzsL | bBXcCμL |
| ##YΔN## | YΔbFwcq | hdMxJtR | hdMxΔtR |
| ##gιj## | zqbQgιZ | DMStbnp | DιStbnp |
| ##FΞK## | ZxΞKMnP | hnZQJYJ | hnZQJΞJ |
| ##sιw## | ZYcLιwC | yPQznXP | yPQzιXP |
| ##gηq## | zηqRLDp | PpJfCmM | PηJfCmM |

S5. Stimuli presented in the semantic control task.

| Probe | Target | Foil | |
| --- | --- | --- | --- |
|  |  | Low control | High control |
| carrot | grape | telescope | bluebell |
| zebra | wolf | pedal | eel |
| mosquito | butterfly | hammock | hawk |
| lighter | lantern | hog | mirror |
| penguin | duck | chocolate | frog |
| daisy | dandelion | trumpet | rose |
| kettle | beaker | berry | tripod |
| revolver | missile | tiger | cigarette |
| hanky | shawl | crocodile | fleece |
| vest | girdle | stork | wallet |
| baton | wand | mink | fork |
| flyer | journal | clam | purse |
| barrel | box | plum | seat |
| lion | puppy | pan | pigeon |
| ivy | thistle | letter | trout |
| apricot | olive | jacket | spinach |
| tomato | peach | gem | cereal |
| algae | bush | hook | moth |
| willow | maple | frost | deer |
| panther | poodle | saxophone | walrus |
| lynx | beagle | wire | ant |
| swan | quail | accordion | ape |
| turtle | snail | palette | calf |
| wasp | dove | outhouse | herring |
| porch | cellar | bulldog | cavern |
| kilt | stocking | seed | ring |
| turban | hood | cedar | shield |
| arrow | bomb | pineapple | pipe |
| corkscrew | pliers | rice | pen |
| mop | hammer | skin | bin |
| clock | compass | walnut | bowl |
| diamond | gravel | eyeball | cork |
| canyon | quarry | elf | dome |
| lake | canal | fly | rain |
| tornado | snow | pup | bubble |
| hurricane | rainbow | leaf | metropolis |
| microwave | screen | kitten | carpet |
| bat | racket | doctor | plank |
| painting | newspaper | thorn | cushion |
| television | phone | root | microscope |
| pebble | brick | oyster | asbestos |
| fan | iron | tree | pillow |
| cleaver | razor | tongue | broom |
| dart | pin | clover | bandage |
| vinegar | bourbon | bone | morphine |
| bracelet | overcoat | flea | sheet |
| flag | blanket | lettuce | tobacco |
| knife | hatchet | birch | hose |
| wing | arm | engine | neck |
| newt | alligator | lightning | perch |
| vault | cave | ewe | chassis |
| pumpkin | raspberry | fountain | sycamore |
| horn | tooth | fence | bark |
| kiwi | cherry | locker | fungus |
| orange | pepper | wicker | shrub |
| kangaroo | bunny | aerial | geese |
| donkey | boar | landscape | banana |
| peacock | lark | typhoon | mackerel |
| prune | bean | poker | creeper |
| onion | chestnut | chisel | pine |
| turkey | crow | violin | mussel |
| ox | seal | linen | carp |
| shrew | mite | grate | pear |
| cod | catfish | lawn | mermaid |
| lavender | moss | wig | fawn |
| wheat | garlic | trophy | nectar |
| blazer | apron | foal | towel |
| syrup | gravy | ankle | beef |
| mould | hedge | piano | lemon |
| whale | toad | shelf | vegetable |
| albatross | wren | uniform | tortoise |
| rhinoceros | goat | rug | crab |
| antelope | mule | capsule | fowl |
| mice | hare | abyss | fruit |
| badger | cattle | guitar | cabbage |
| porpoise | shrimp | fog | weed |
| lord | king | cradle | infant |
| sailor | driver | mural | owner |
| mayor | emperor | syringe | dancer |
| painter | pianist | amethyst | professor |
| magician | clown | cliff | jockey |
| buckle | button | thumb | piston |
| taxi | ship | blossom | propeller |
| digger | crane | hound | jet |
| university | hospital | insect | shed |
| pew | couch | mouth | toilet |
| mountain | pyramid | doe | arch |
| cottage | cabin | thigh | belfry |
| office | bank | nutmeg | prison |
| fisherman | hunter | oil | teacher |
| spear | grenade | secretary | tablespoon |
| ambulance | caravan | bacteria | plane |
| sofa | stool | skull | desk |
| plumber | builder | page | actress |
| rocket | van | rogue | typewriter |
| farmyard | allotment | sergeant | coast |
